# Indel driven rapid evolution of core nuclear pore protein gene promoters

**DOI:** 10.1038/s41598-023-34985-0

**Published:** 2023-05-17

**Authors:** David W. J. McQuarrie, Adam M. Read, Frannie H. S. Stephens, Alberto Civetta, Matthias Soller

**Affiliations:** 1 School of Biosciences, College of Life and Environmental Sciences, University of Birmingham, Edgbaston, Birmingham, B15 2TT UK; 2 Birmingham Centre for Genome Biology, University of Birmingham, Edgbaston, Birmingham, B15 2TT UK; 3 Department of Biology, University of Winnipeg, Winnipeg, MB R3B 2E9 Canada

**Keywords:** Evolution, Evolutionary genetics, Molecular evolution, Phylogenetics, Speciation

## Abstract

Nuclear pore proteins (Nups) prominently are among the few genes linked to speciation from hybrid incompatibility in *Drosophila*. These studies have focused on coding sequence evolution of Nup96 and Nup160 and shown evidence of positive selection driving nucleoporin evolution. Intriguingly, channel Nup54 functionality is required for neuronal wiring underlying the female post-mating response induced by male-derived sex-peptide. A region of rapid evolution in the core promoter of *Nup54* suggests a critical role for general transcriptional regulatory elements at the onset of speciation, but whether this is a general feature of Nup genes has not been determined. Consistent with findings for *Nup54*, additional channel Nup58 and Nup62 promoters also rapidly accumulate insertions/deletions (indels). Comprehensive examination of Nup upstream regions reveals that core Nup complex gene promoters accumulate indels rapidly. Since changes in promoters can drive changes in expression, these results indicate an evolutionary mechanism driven by indel accumulation in core Nup promoters. Compensation of such gene expression changes could lead to altered neuronal wiring, rapid fixation of traits caused by promoter changes and subsequently the rise of new species. Hence, the nuclear pore complex may act as a nexus for species-specific changes via nucleo-cytoplasmic transport regulated gene expression.

## Introduction

**Figure 1:**
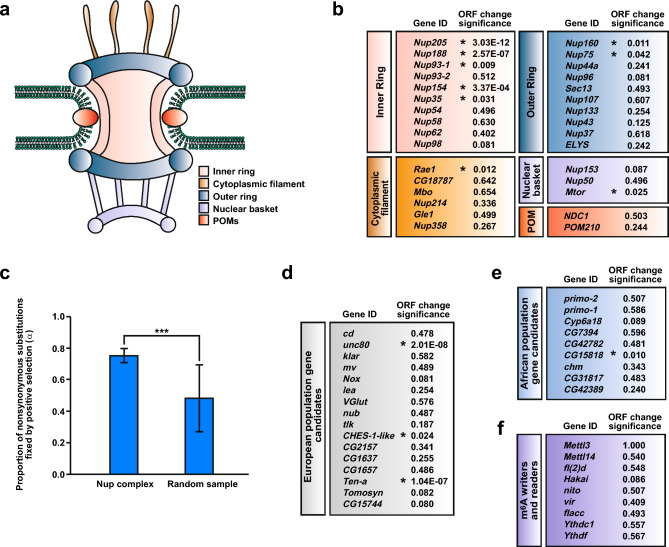
Positive selection drives the evolution of the nuclear pore complex proteins. (**a**) Graphic depiction of the nuclear pore complex with differentially coloured subcomplexes inner ring (IR, pink), outer ring (OR, blue), cytoplasmic filament (CF, orange), nuclear basket (purple) and pore membrane proteins (POMS, red) embedded in the nuclear envelope lipid-bilayer (green). (**b**) Significance of synonymous to non-synonymous changes in Nup subcomplex open reading frames (ORFs) indicated by an asterisk and p values. (**c**) Average proportion of substitutions fixed by positive selection (α) shown with standard deviation. 30 genes were randomly sampled 100 times with replacements from the list of Nups and from the genome. Statistically significant differences from unpaired student t tests are indicated by asterisks (*** p ≤ 0.0001). (**d–f**) Significance of synonymous to non-synonymous changes in ORFs indicated by an asterisk and p values. As positive control groups the "best candidate genes" under balancing selection for European (**d**) and African (**e**) populations were used. As a negative control group, the highly conserved m^6^A writer complex and m^6^A readers were analysed (**f**).

The nuclear pore protein complex (Nup complex) provides a physical barrier between the nucleus and cytoplasm requiring active transport for cargos above about 40 kDa^1^. The Nup complex consists of 30 proteins conserved in *Drosophila* (Fig. 1), which are grouped into subcomplexes termed outer ring (OR), inner ring (IR), cytoplasmic filaments (CF), nuclear basket (NB) and pore membrane proteins (POMS). The transport channel is made up of three phenylalanine-glycine-rich (FG) repeat domains of Nup54, Nup58 and Nup62. The Nup complex can contribute to differential expression of other genes as e.g. actively transcribed genes can be in the proximity of the Nup complex^2–5^.

Male derived substances transferred during mating change female physiology and behaviour to guarantee reproductive success in many insects^6–9^. Rapid divergence in male versus female interactive molecules can hamper reproductive success, potentially leading to the establishment of new species for example by compromising fertility or viability among hybrids^10–18^. Such hybrid incompatibility among closely related *Drosophila* species has been used to identify responsible genes. Among the few genes identified were nuclear pore proteins Nup96 and Nup160^19–22^. Interestingly, Nups are overrepresented among speciation genes^19^. In particular, Nup96 and Nup160 have been shown to be under adaptive evolution and at an individual gene level can cause hybrid lethality, contrasting with reports of multiple linked factors being necessary for hybrid lethality^19–21^.

Sex peptide is the master regulator of the female post-mating response in *Drosophila melanogaster*^8,23^. A screen for sex-peptide insensitive mutants identified Nup54 functionality to be important for neuronal wiring of circuits involved in regulating post-mating behaviours^15^. In particular, a deletion in the promoter of the *Nup54* gene has been associated with altered nucleo-cytoplasmic shuttling in eight *pickpocket* (*ppk)* expressing neurons in the central brain leading to wiring defects and a compromised female post-mating response directed by male-derived sex-peptide transferred during mating^15^. Moreover, this *Nup54* promoter deletion allele maps to a region for rapid evolution in the *Nup54* promotor suggesting sexual conflict driving female escape from male manipulation by sex-peptide under unfavourable conditions^15^. Furthermore, channel Nups have also been attributed a role in transposon silencing in the germline by facilitating processing of short piRNAs from long pre-curser RNAs of the *flamenco* locus, which functions as a ‘master off switch’ for transposons^24^, indicating that the disruption to Nup complex regulation likely has pleiotropic effects.

Given prior findings about adaptive evolution of the Nup96 and Nup160 coding region^19–22^, we systematically extended this analysis to all Nups. Moreover, since the *Nup54* gene promoter evolves rapidly we systematically analysed all Nup gene promoter regions. Using *Drosophila* phylogenomics we reveal that promoters of core Nup complex genes are accumulating insertions/deletions (indels) driving rapid evolution. These findings suggest that the nuclear pore complex is a nexus for species-specific changes via nucleo-cytoplasmic transport regulated gene expression.

## Results

### Positive selection drives the evolution of nuclear pore complex proteins

To assess whether the protein coding region (Open Reading Frame, ORF) of Nups were under selection, we performed McDonald-Kreitman tests (MKTs) to compare the number of polymorphisms in the *D. melanogaster* ancestral Congo population, and divergence between *D. melanogaster* and its closest relative *D. simulans*. This analysis showed positive selection for only nine Nup ORFs (Fig. 1b). Moreover, nucleoporins have a higher proportion of nonsynonymous substitutions fixed by positive selection (α) (Fig. 1c, Supplementary Dataset 1)^25^, meaning stronger evidence of positive selection, than expected from a random genome sample of equal size.

To substantiate the significance of the Nup complex subcomplex MKT results, we carried out the same analysis for additional sets of genes that either evolve rapidly or are highly conserved. We used two positive control groups identified in Croze et al. (2017)^26^ as the "best candidate genes" under balancing selection for European (Fig. 1d) and African (Fig. 1e) populations. As a negative control group we analysed the m^6^A mRNA methylation machinery (Fig. 1f, writer complex: Mettl3, Mettl14, fl(2)d, virilizer, flacc, nito and Hakai; readers: YTHDC1 and YTHDF), because high evolutionary conservation is required to maintain complex stoichiometry to guarantee functionality making this group optimal for use as a control group to monitor protein evolution (Fig. 1f)^27–31^. Interestingly, the two positive control groups had a low number of rapidly evolving members for both the European (three out of sixteen) and African (one out of nine) groups (Fig. 1d, e). As expected, all m^6^A group members followed the neutral hypothesis (Fig. 1f). Taken together, these results demonstrate that Nup complex members are fast evolving, particularly the inner ring subcomplex (Fig. 1b).

### Promoter regions of channel Nups 58 and 62 have diverged in closely related species

**Figure 2:**
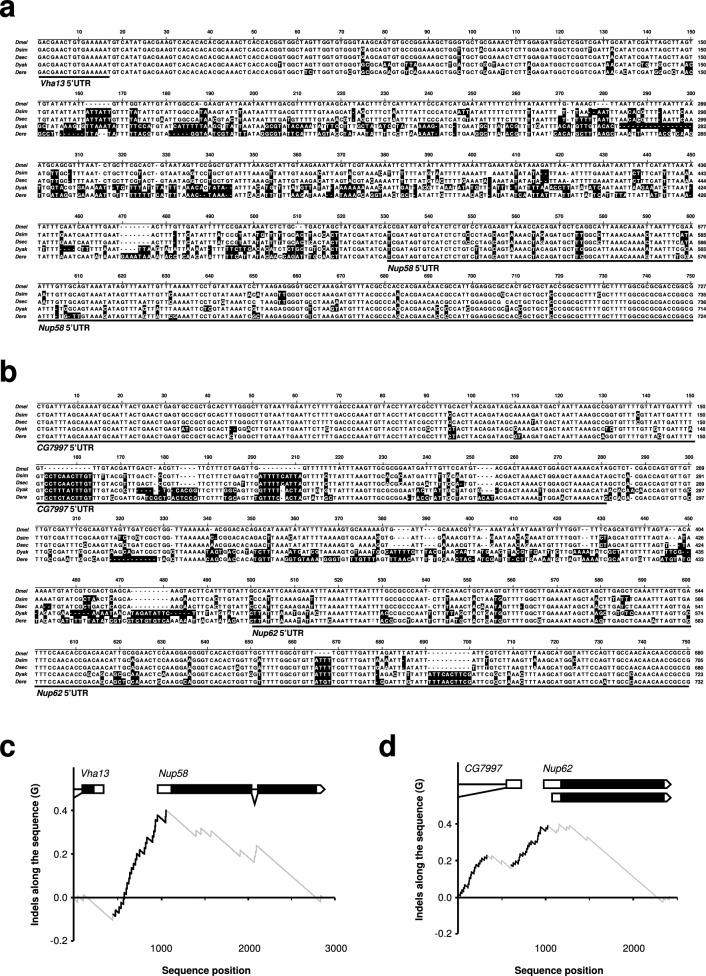
Promoter regions of channel Nups 58 and 62 have diverged in closely related species. **(a,b)** Sequence alignment of the *Nup58* and *Nup62* promoter regions from closely related species. Nucleic acids changes from *D. melanogaster* are indicated in black. Transcribed parts of the *Vha13* and *CG7997* 5′UTR and the *Nup58* and *Nup62* 5′UTR are indicated by a line. (**c,d**) Plot of cumulative differences along the sequence (G) between the relative occurrences of indels and their position from the alignment of the gene region around *Nup58* and *Nup62* between *D. melanogaster*, *D. simulans*, *D. sechellia*, *D. yakuba* and *D. erecta*. Positions in the alignment with significant stretches of substitutions are identified by black line(s).

Since we previously identified the promoter of the channel Nup54 as a region of rapid sequence divergence^15^, we examined the promoters of the other two channel Nups 58 and 62 among closely related species of *Drosophila* (*D. melanogaster*, *D. simulans*, *D. sechellia*, *D. yakuba* and *D. erecta*) (Fig. 2). This detailed analysis revealed that also the promoters of the other two channel Nups accumulated indels rapidly (Fig. 2). Moreover, these changes contain a number of indels (Fig. 2c, d) that can fundamentally impact on transcription factor binding commonly found in the proximal region before the TATA box^32–36^.

Next, we extended this detailed analysis to Nup98-96 and Nup160, since these have been implicated as drivers of speciation^19^. As with Nup58 and Nup62, this analysis indicated divergence in promoter regions of the two genes (Supplementary Fig. 1a, b) and highlighted accumulation of indels in promoters of Nup98-96 and Nup160 (Supplementary Fig. 1c, d).

### Inner and outer ring nuclear pore protein gene subcomplexes undergo rapid evolution through indel accumulation in promoters

**Figure 3:**
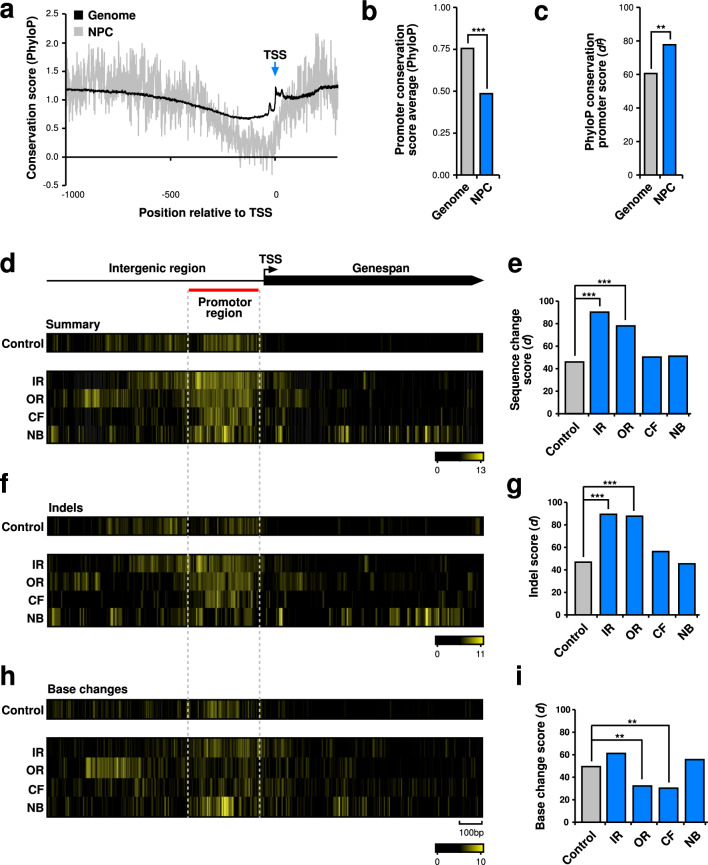
Core nuclear pore protein genes rapidly evolve through indel accumulation in promoters. **(a)** Evolutionary conservation of nucleotide positions around the TSS depicted by PhyloP27way conservation score averages for the *D. melanogaster* genome (black) and the Nup complex (grey). (**b**) Evolutionary conservation measured by PhyloP27way conservation score averages for the 350 nucleotide promoter regions compared between the Nup complex and the genome. Statistically significant differences from unpaired student t tests are indicated by asterisks (*** p ≤ 0.0001). **(c)** Evolutionary conservation measured by PhyloP27way conservation is shown as promoter *d*^*P*^ scores for the 350 nucleotide window in the promoters of the Nup complex and the genome averages. Statistically significant differences from non-parametric chi-squared tests are indicated by asterisks (** p ≤ 0.001). (**d–i**) Heatmaps indicating divergence for all changes (**d**), indels (**f**), and base changes (**h**) in yellow among closely related *D. melanogaster*, *D. simulans*, *D. sechellia*, *D. yakuba* and *D. erecta* for the control group of genes (m^6^A writer complex and readers) and the inner ring (IR), outer ring (OR), cytoplasmic filament (CF) and nuclear basket (NB) below the gene model with the TSS indicated by an arrow. The red line indicates the promoter region used for quantification of the substitution rate. Quantification of the change rate of all changes (**e**), indels (**g**), and base changes (**i**) in the TATA box distal region (from −30 to −380). Statistically significant differences from non-parametric chi-squared tests are indicated by asterisks (**p ≤ 0.001, ***p ≤ 0.0001).

Next, we examined the promoter region of all remaining Nups in the closely related *Drosophila* species to see whether accumulation of mutations is a general feature of the promoters of this class of genes (Fig. 3). We analysed conservation in 27 insect species through PhyloP27way data^37,38^. To determine whether the Nup complex evolves rapidly compared to the genome, we used PhyloP scores as a measure of conservation upstream of the transcription start site (TSS) (Fig. 3a). We observed a significant decrease in Nup complex sequence conservation in the promoter region upstream of the predicted TATA box site (−30 to −380), where the average PhyloP conservation score was significantly lower for the Nup complex compared to the genome (Fig. 3a, b). Additionally, accumulation of changes in promoter regions were analysed as PhyloP sliding window diversity scores (*d*^*P*^) and compared between the Nup complex and the genome (Fig. 3c). Here, the Nup complex significantly accumulated sequence changes compared to the genome (Fig. 3c).

To focus more specifically on *Drosophila*, we analysed promoter evolution between species in the *melanogaster* subgroup^39^. As a control group we used the m^6^A mRNA methylation machinery control group again because they also have similar TSS properties to Nup complex genes comparative to other gene groups like ribosomal genes^40,41^, making this group optimal for use as a control group to monitor promoter evolution^27–30^.

We analysed accumulation of changes in promoter regions using sliding window diversity scores (*d*) and compared scores between Nup complex subcomplexes and the control group. As has been observed for *Drosophila* and human promoters^36,40,42^, the region immediately before the TATA box constitutes transcription factor binding sites (−30 to −380), and showed a slightly increased rate of sequence changes in m^6^A mRNA methylation writer complex and readers consistent with the general trend of greater sequence diversity upstream of gene TSSs (Fig. 3d–i). We then analysed the promoters of the different Nup sub-complexes IR, OR, CF and NB. We investigated all sequence differences (Fig. 3d, e) and performed a more detailed analysis split into indels (Fig. 3f, g) and base changes (Fig. 3h, i). This analysis revealed that the core nuclear complex consisting of the IR and OR genes showed a significantly enhanced rate of indel driven evolution compared to the control group or the CF and NB group of genes (Fig. 3f, g). Analysis of all sequence differences mirrored the indel analysis including a significant increase in the number of differences in IR and OR Nups (Fig. 3d, e). In contrast base changes were reduced for OR and CF Nups (Fig. 3h, i).

## Discussion

Here, through a combined analysis of polymorphism and divergence between closely related *Drosophila* species we show that the evolution of Nup complex proteins is driven by positive selection. These results are in agreement with previous findings of adaptive evolution of Nups^19^. Our MKT results support previously reported evidence of positive selection in these same species for Nup160, with only marginally significant evidence for Nup96^20,21^. Slight differences in results are not unexpected when different source populations are used for the analysis^16^.

Focussing on upstream and downstream Nup gene regions, we reveal a bias for inclusion of nucleotide changes in the core members of the Nup complex. Consistent with channel *Nup54*^15^, further analysis of upstream regions of the other channel Nups 58 and 62 reveals that their promotor regions rapidly accumulate indels. Through examination of promoter regions of Nup subcomplex members, we identify the core IR and OR Nups as rapidly accumulating indels. Here, the core Nup complex members likely act as drivers of species-specific variation through indel driven changes in promoters, subsequently modifying regulation of nucleo-cytoplasmic transport regulated gene expression.

Although the megadalton Nup complex has mainly been associated with providing a physical barrier between nucleus and cytoplasm, recent discovery of pleiotropic functions of channel Nup54 provide new insights how the Nup complex could drive speciation^15^. Nup54 is required for neuronal wiring underlying the female post-mating response induced by male-derived sex-peptide. Although we have not observed an obvious effect of incompatibility of Nup54 through sterility or lethality, this could be extended to be incompatibility resulting in incompatible behaviours. Considering the involvement of Nup54 in mating behaviour, one plausible scenario could involve exchange of sexual conflict for intragenomic conflict as the adaptive driver of initial divergence^10,13–15,18,43^. Here, the *Nup54* gene promoter could undergo rapid changes driven by sexual conflict. If this occurs in one or more isolated populations, pleiotropic effects of changes to Nup54 regulation of neuronal wiring, sexual differentiation or transposon regulation could result in genetic incompatibilities when the isolated population(s) come into contact with the original population^15^.

Alternatively, Nups could drive speciation through their regulation of transposon silencing directly. Channel Nups are required for the generation of piRNAs originating from the *flamenco* locus in the germline to silence transposons^24^. Maternally inherited piRNAs are essential to transposon silencing and an imbalance can lead to a phenomenon called hybrid dysgenesis, imposing reduced fecundity in the female offspring when the male genome contributes novel transposable elements to be silenced^44^. Key to silencing are epigenetically inherited piRNAs from the female to prime the “ping-pong” cycle for amplification to increase the silencing capacity by heterochromatinization for preventing transposon mobilisation^44–47^. Compromised transposon silencing leading to deleterious reductions of fecundity could be escaped by altered Nup complex function. Such changes likely trigger pleiotropic effects that could include changes in neuronal wiring and behaviour. Such changes, however, could become fixed as a result of behavioural isolation, but restoring piRNA silencing regulation to its original state would not revert to the initial neurological state. In essence, if escape from lower fecundity as a result of hybrid dysgenesis^44–47^, leading to reduced transposon silencing is coupled to changes in the morphology or behaviour and behavioural isolation, new species could be established very quickly. In particular, if changes in Nup functionality result in rewiring of neuronal circuits as indicated for Nup54, behavioural preferences leading to rapid isolation are likely. In fact, mate preference has changed in *D. simulans* through rewiring of sensory neuron projections to fruitless P1 neurons that control courtship^48^.

## Conclusion

Changes in gene expression have profound effects during species divergence and phenotypic adaptation, and such changes can lead to hybrids’ gene mis-expression and dysfunction^49–53^. The molecular mechanism how new species arise through differential regulation of gene expression remains uncertain. The newly discovered role of channel Nups in piRNA processing in the germline to maintain transposon silencing provokes the claim that any compensation of the negative impact on fecundity from hybrid dysgenesis would be favoured. However, if as result of the pleiotropic effect of Nups changes in neuronal wiring and behaviour occur, these changes could be irreversible if behavioural isolation has already advanced^15,24^. Since mutations in promoters can directly affect gene expression regulation^54–56^, such changes could be rapidly fixed in contrast to recessive changes in coding regions, which would remain hidden in heterozygosity. Our systematic analysis of the evolution of all Nups coding and promoter regions suggests a mode of evolution through changes of sequences upstream of the TSS, particularly in the promoters of core Nups, and supports the possibility that compensation of deleterious changes in the germline can lead to altered neuronal wiring and rapid fixation of adaptive traits.

## Materials and methods

### Open reading frame analysis

To determine whether the ORFs of nucleoporins are under selection, the PopFly online database (imkt.uab.cat) developed from the Drosophila Genome Nexus project assembling sequence data of around 1100 *D. melanogaster* genomes were used to perform MKTs to analyse polymorphism data from the ancestral Congo population because it is a sub-Saharan population with higher ancestral stability than other populations^57–59^. Synonymous and non-synonymous polymorphisms within *D. melanogaster* or between *D. melanogaster* and *D. simulans* were obtained and significance determined by Fisher’s Exact Test, with significance defined as p ≤ 0.05 after FDR correction. We used the concatenate and the compare against whole-genome distribution advanced options within the PopFly database analysis tool to compare the proportion of substitutions fixed by positive selection (α). We compared the 30 Nups against a random sample of the same size by randomly sampling from both datasets 100 times. Significance was calculated through an unpaired t test with significance defined as p ≤ 0.05.

### Sequence/data retrieval and alignment

Nucleoporin gene and promoter sequences for the five analysed *Drosophila* species (*D. melanogaster*, *D. simulans*, *D. sechellia*, *D. yakuba*, and *D. erecta*) were retrieved from UCSC genome browser (genome.ucsc.edu) using the Table Browser tool^37,38^. Pairwise and multiple alignments were carried out with clustalW using the alignment program version MEGA11 default alignment settings^60^. PhyloP27way data were sourced from UCSC genome browser through the Table Browser tool^37,38^. Data points were collected for a region of 1000 nucleotides upstream and 300 nucleotides downstream of gene TSSs relative to their transcription strands.

### Accumulation of substitutions along extended gene regions

To test for nonrandom accumulation of indels along the Nup extended gene regions between the five analysed *Drosophila* species (*D. melanogaster*, *D. simulans*, *D. sechellia*, *D. yakuba*, and *D. erecta*) we determined significant deviations from a uniform distribution of substitutions using an empirical cumulative distribution function, as described by Civetta et al. (2016)^61^. The position of the indel event was defined as the 5’ site of the start of the indel in the alignment ^62^. The function (G) detects monotonic increases in substitutions (n) measured as the difference between the relative occurrence of a nucleotide change and its relative position in the alignment^61^. Whether differences between the values of the G function (ΔG) between substitutional events deviates from a random accumulation of changes is tested using Monte Carlo simulations to produce 100,000 samples of n events by sampling sites without replacement along the alignment^61^.

### Comparison of substitution rates

To analyse the promoters of Nups and the control group of the m^6^A writer complex and readers, a region of 1000 nucleotides upstream and downstream of the TSS was used with the TSS as an anchor point. Regions were aligned between the five analysed *Drosophila* species (*D. melanogaster*, *D. simulans*, *D. sechellia*, *D. yakuba*, and *D. erecta*) and alignments were translated into events^63^. Per Tang and Lewontin (1999)^63^, 0 signified no sequence difference between all analysed species and 1 signified comparative sequence divergence for ≥ 1 species. The same was also performed for indels and base changes individually, where 0 signified no indel or base change event and 1 signified an indel or base change event for ≥ 1 species. A sliding window of five nucleotides upstream and downstream of each position was summed and processed to define sliding event (*Se*) scores which were used to generate heatmaps. *Se* scores were averaged between the different Nup subcomplexes and the m^6^A control. To calculate sequence change accumulation (percentage of events greater than the average control promoter sliding window score (*d*)), a 350 nucleotide region upstream of the approximated TATA box region was taken. The total number of *Se* scores greater than the average control *Se* (*Se*^*C*^) was divided by the total number of events in the region (*N*).

$$d = \frac{{\text{Total\;number\;of\;Se\;events\;where}\;Se > Se^{C} }}{N} \times {{100}}$$

Significance was defined by non-parametric chi-squared tests versus the control score with one degree of freedom, where the percentage groupings were > *Se*^*C*^ (*d*) and ≤ *Se*^*C*^. P values ≤ 0.05 with Bonferroni correction were deemed statistically significant.

To compare the conservation rate of the Nup complex compared to the *D. melanogaster* genome, PhyloP27way scores for a region of 1000 nucleotides upstream and 300 nucleotides downstream of the TSS for all *Drosophila* genes was performed. Genes without data points for the full 1300 nucleotide region were omitted. Genome-wide and Nup complex mean PhyloP scores were calculated using R version 4.4.2^64^. The average 350 nucleotide region upstream of the approximated TATA box region was compared between the two and significance was calculated using an unpaired student t-test. P values ≤ 0.05 were deemed statistically significant. For the same region of the Nup complex and genome average PhyloP *d* (*d*^*P*^) was calculated, and significance determined as follows: the total number of PhyloP (*p*) scores in the 350 nucleotide regions less than the control group average promoter region (*p*^*C*^) replaced the total number of *Se* events where *Se* > *Se*^*C*^ (see below).

$$d^{P} = \frac{{\text{Total\;number\;of}\;p\;{\text{scores }}< p^{C} }}{N} \times {{100}}$$

Significance was defined by non-parametric chi-squared tests versus the control score with one degree of freedom, where the percentage groupings were < *p*^*C*^ (*d*^*P*^) and ≥ *p*^*C*^. P values ≤ 0.05 were deemed statistically significant.

## Supplementary Information

Supplementary Figure 1.

Supplementary Information.

## Data Availability

All data generated or analysed during this study are included in Supplementary Dataset 1.
