# Supplementary material for: Indel driven rapid evolution of core nuclear pore protein gene promoters
Source: Sci Rep. 2023 May 17;13:8035. doi: 10.1038/s41598-023-34985-0 (PMC10192361; doi:10.1038/s41598-023-34985-0)

Supplementary Materials for

Indel driven rapid evolution of core nuclear pore protein gene promoters

DAVID W. J. MCQUARRIE[1,2*], ADAM M. READ[1*], FRANNIE H. S. STEPHENS[1], ALBERTO CIVETTA[3,4] AND MATTHIAS SOLLER[1,2,4]

Supplementary Figures

Supplementary Figure 1: Promoter regions of outer ring Nups 98-96 and 160 have diverged in closely related species.

a, b) Sequence alignment of the *Nup98-96* and *Nup160* promoter regions from closely related species. Nucleic acids changes from *D. melanogaster* are indicated in black. Transcribed parts of the *mbc* and *Csl4* 5′UTR and the *Nup98-96* and *Nup160* 5′UTR are indicated by a line.

c, d) Plot of cumulative differences along the sequence (G) between the relative occurrences of indels and their position from the alignment of the gene region around *Nup98-96* and *Nup160* between *D. melanogaster*, *D. simulans*, *D. sechellia*, *D. yakuba* and *D. erecta*. Positions in the alignment with significant stretches of substitutions are identified by black line(s).

Supplementary Figure1

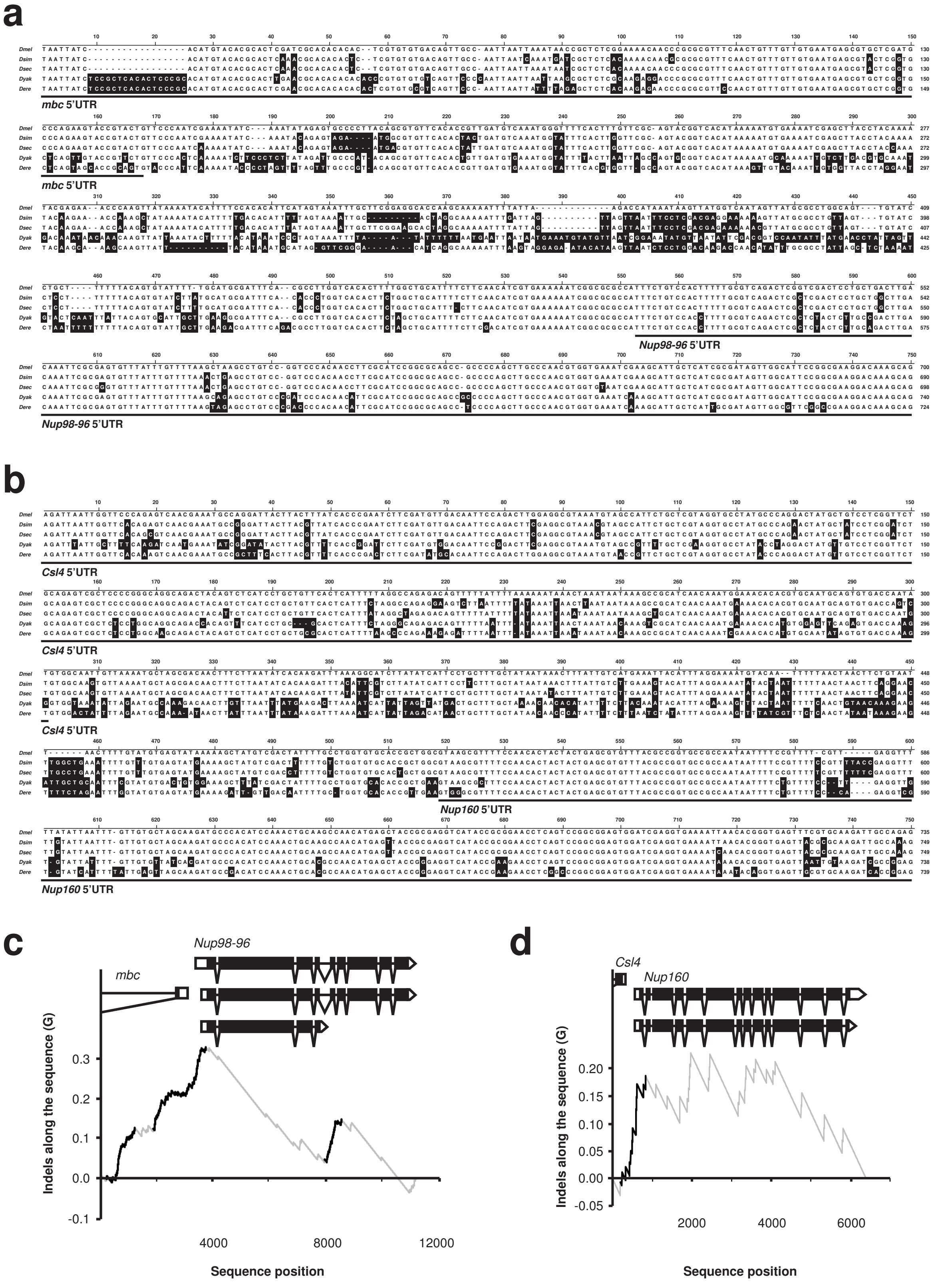

Supplement: Supplementary file 1 — Supplementary Figure 1. [file 41598_2023_34985_MOESM1_ESM.pdf]
